# Science, Policy, and the Transparency of Values

**DOI:** 10.1289/ehp.1408107

**Published:** 2014-03-25

**Authors:** Kevin C. Elliott, David B. Resnik

**Affiliations:** 1 Lyman Briggs College, Department of Fisheries and Wildlife, and Department of Philosophy, Michigan State University, East Lansing, Michigan, USA; 2 National Institute of Environmental Health Sciences, National Institutes of Health, Department of Health and Human Services, Research Triangle Park, North Carolina, USA

## Abstract

Background: Opposing groups of scientists have recently engaged in a heated dispute over a preliminary European Commission (EC) report on its regulatory policy for endocrine-disrupting chemicals. In addition to the scientific issues at stake, a central question has been how scientists can maintain their objectivity when informing policy makers.

Objectives: Drawing from current ethical, conceptual, and empirical studies of objectivity and conflicts of interest in scientific research, we propose guiding principles for communicating scientific findings in a manner that promotes objectivity, public trust, and policy relevance.

Discussion: Both conceptual and empirical studies of scientific reasoning have shown that it is unrealistic to prevent policy-relevant scientific research from being influenced by value judgments. Conceptually, the current dispute over the EC report illustrates how scientists are forced to make value judgments about appropriate standards of evidence when informing public policy. Empirical studies provide further evidence that scientists are unavoidably influenced by a variety of potentially subconscious financial, social, political, and personal interests and values.

Conclusions: When scientific evidence is inconclusive and major regulatory decisions are at stake, it is unrealistic to think that values can be excluded from scientific reasoning. Thus, efforts to suppress or hide interests or values may actually damage scientific objectivity and public trust, whereas a willingness to bring implicit interests and values into the open may be the best path to promoting good science and policy.

Citation: Elliott KC, Resnik DB. 2014. Science, policy, and the transparency of values. Environ Health Perspect 122:647–650; http://dx.doi.org/10.1289/ehp.1408107

## Introduction

A recent news story in *Nature* (Cressey 2013) reported on a heated dispute between opposing groups of scientists in response to an alleged but uncited draft report by the European Commission (EC) on its proposed regulatory policy concerning endocrine disrupting chemicals (EDCs) (EC 2013; Horel and Bienkowski 2013). One of the participants in the dispute, Andrea Gore, a professor at the University of Texas at Austin and editor of the journal *Endocrinology,* claimed that this was “possibly the most remarkable experience in my career” and that it was “definitely more confrontational than most scientists are used to” (Cressey 2013). Although the details of the dispute revolve around questions about how to identify and regulate EDCs, it also highlights broader issues about how scientists should communicate with policy makers in a manner that is both policy relevant and appropriately objective. Some of the participants in the dispute called for making a sharper distinction between science and policy (Bergman et al. 2013), but we argue here that society is better served when scientists strive to be as transparent as possible about the ways in which interests or values may influence their reasoning.

The conflict erupted when a group of 18 journal editors published an editorial in *Food and Chemical Toxicology* accusing the EC of preparing a regulatory system for EDCs that is “based on virtually complete ignorance of all well-established and taught principles of pharmacology and toxicology” (Dietrich et al. 2013). The editorial and an accompanying letter (Dietrich et al. 2013) focused on two issues: First, the authors of the editorial criticized the EC for proposing a system in which evidence of endocrine disruption obtained in animals and various other experimental systems would be presumed to be relevant to humans in the absence of evidence to the contrary. Second, the authors expressed the concern that the EC would presume that EDCs do not have a threshold dose below which they cease to induce adverse effects.

In response to this initial editorial, several subsequent commentaries were published (Bergman et al. 2013; Gore et al. 2013; Grandjean and Ozonoff 2013). An editorial signed by 41 scientists and published in *Environmental Health* (Bergman et al. 2013) questioned whether the EC actually envisioned a regulatory policy with the characteristics described by Dietrich and his co-authors. The editorial by Bergman et al. (2013) also pointed out that the International Programme on Chemical Safety (IPCS) Framework document for risk assessment (IPCS 2002) adopts a default assumption that evidence of toxicity in animals is relevant to humans. Moreover, the editorial argued that evidence for the existence of thresholds for EDCs, especially at the population level, remains inconclusive. Another editorial, published in the journal *Endocrinology* (Gore et al. 2013), emphasized that the assumption of no threshold for the adverse effects of EDCs is reasonable, given the evidence.

## A Dispute about Science Communication

A look beyond the scientific details of the case shows that these and other editorials (Bergman et al. 2013; Gore et al. 2013; Grandjean and Ozonoff 2013; Horel and Bienkowski 2013; Lehman-McKeeman and Kaminski 2013) have raised significant issues about how scientists can appropriately inform public policy. The response in *Environmental Health* states that “[t]he most worrying aspect of the editorial by Dietrich et al. is the blurring of the border between what constitutes science and what belongs to the realm of political, societal and democratic choices” (Bergman et al. 2013). This concern is clearly expressed in the title of the editorial: “Science and policy on endocrine disrupters must not be mixed.” Related to this worry about mixing policy with science, an editorial by Grandjean and Ozonoff (2013) suggested that a crucial flaw in the editorial by Dietrich et al. (2013) was its failure to include a conflict-of-interest disclosure.According to Grandjean and Ozonoff, trust is necessary among scientists, editors, publishers, and members of the public, and that trust is broken when authors do not acknowledge their competing interests.

These editorials (Bergman et al. 2013; Grandjean and Ozonoff 2013) highlight the worry that scientists are in danger of losing their objectivity when they wade into the policy domain. Because objectivity is one of science’s most important goals, this concern has considerable merit. Even when scientists do not actually compromise their objectivity, people may perceive that they have done so, which can undermine the public’s trust in science. Although engaging in policy-relevant research can threaten science’s objectivity, a great deal would be lost if scientists refused to enter these waters because public policies should typically be informed by the best available scientific evidence (Pielke 2007; Resnik 2009). In a commentary that cites Dietrich et al. (2013) approvingly, Lehman-McKeeman and Kaminski (2013) argued that the Society of Toxicology (SOT) “must avoid playing it safe.” In other words, they call for the members of the SOT to inform policy makers about issues on which they have expertise. However, if toxicologists and other scientists are to help inform policy, they face the question of how to do this without losing their objectivity or the public’s trust.

## Discussion

In the past, scientists and philosophers have argued that the best way to maintain science’s objectivity and the public’s trust is to draw a sharp line between science and human values or policy (Longino 1990). However, it is not possible to maintain this distinction, both because values are crucial for assessing what counts as sufficient evidence and because ethical, political, economic, cultural, and religious factors unavoidably affect scientific judgment (Douglas 2009; Elliott 2011; Longino 1990; Resnik 2007, 2009). Insisting that science is value-free, when the arguments and evidence show that this is an unrealistic goal, perpetuates a misunderstanding that interferes with the public’s understanding of the scientific process and may, paradoxically, undermine the public’s trust in science. We suggest that society is likely to be better served when scientists strive to be as transparent as possible about the ways that interests and values may influence their judgment and reasoning, while still striving for objectivity. Transparency can promote public trust by helping laypeople understand how both empirical evidence and value assumptions enter into scientific decision making and policy formation. As the National Research Council (NRC) report *Understanding Risk* (NRC 1996) emphasized, it is usually unrealistic to keep the process of risk characterization purely value-free. Instead, the report called for incorporating broad-based deliberation about the values that inform risk assessments in order to provide a context for the scientific analyses that are part of the assessment process.

*Standards of evidence*. The first reason it is problematic to draw a sharp separation between science and values is that values are necessary to decide what standards of evidence to demand when informing policy decisions (Ashford 1988; Cranor 1993; Douglas 2009; Elliott 2011). Several comments from the recent dispute about the proposed EC policy illustrate the difficulties of trying to ignore this necessary role for values. First, as noted earlier, the editorial that calls for science and policy on EDCs to remain “unmixed” (Bergman et al. 2013) insists that it is reasonable to assume that evidence of toxicity in animals is relevant to humans. The authors argue that the alternative assumption (i.e., that effects in animals do not provide evidence for effects in humans) “would be unworkable” (Bergman et al. 2013). But this conclusion incorporates value judgments concerning the standards of evidence that are appropriate for regulating chemicals. Insisting that chemicals should be regulated only in response to evidence from human studies would help to prevent false positive conclusions about chemical toxicity, but it would also prevent society from taking effective action to minimize the risks of chemicals before they produce measurable adverse effects in humans. Moreover, insisting on human studies would result in failure to identify some human health risks because the diseases are rare, or the induction and latency periods are long, or the effects are subtle (Cranor 2011).

Similarly, Gore et al. (2013) argued that “[t]he assumption of no threshold has been widely used, for many years, in the regulation of genotoxic carcinogens, often based upon in vitro data. We believe extending this precedent to EDCs is supported by the science.” But the claim that the no-threshold hypothesis is “supported by the science” depends on implicit assumptions about how much scientific evidence is needed to justify formulating policy on this basis. And the question of how much evidence is needed should depend in part on value judgments about the relative benefits and harms to society of assuming (or not assuming) a threshold when performing risk assessments of EDCs. In this case, past toxicological experience may support the threshold hypothesis, whereas other lines of evidence (such as the proposed molecular mechanisms by which EDCs could disrupt development and generate irreversible effects on endocrine-sensitive organs) support the no-threshold hypothesis. Thus, the dispute between Gore et al. (2013) and Dietrich et al. (2013) regarding the adoption of thresholds for EDCs could be clarified if the participants were more forthcoming about their assumptions regarding the level and kind of evidence needed to justify adopting or rejecting the threshold hypothesis.

In their editorial, Lehman-McKeeman and Kaminski (2013) call for the members of the SOT to be “strong advocates for applying the best science” to policy issues and to craft regulatory policies that are “based on sound science.” Even this seemingly innocuous advice to promote decisions based on good science hides significant value judgments about the appropriate standards of evidence in policy contexts (Ashford 1988; Cranor 1993). If the “best science” and “sound science” are interpreted to mean science that meets the highest standards of scientific evidence, then it is not clear that regulatory policy must always be based on this form of evidence, because it may be appropriate to use different standards of evidence to protect the public from risks. A continuum of different kinds and amounts of evidence could be demanded for policy decisions (Ashford 1988). Very high standards of evidence are typically expected in order to infer causal relationships or to approve the marketing of new drugs. In other social contexts, such as tort law and chemical regulation, weaker standards of evidence are sometimes acceptable to protect the public (Cranor 2008). To demand the very highest standards of evidence for chemical regulation—including, for example, human evidence, accompanying animal data, mechanistic evidence, and clear exposure data—would take very long periods of time and leave the public’s health at risk. Thus, the demand that regulators rely on the same standards of evidence for toxicity as the scientific community uses in other contexts is itself a value-laden proposal.

The value-laden assumptions about standards of evidence in this dispute over endocrine disruption are similar to broader social disputes over the precautionary principle (Kriebel et al. 2001; Martuzzi 2007; Miller and Conko 2001; Sunstein 2005). Indeed, the title of the editorial by Dietrich et al. (2013) begins with the claim, “Scientifically unfounded precaution drives European Commission’s recommendations on EDC regulation.” Some critics of the precautionary principle, such as Dietrich and his coauthors, argue that precaution runs counter to scientific principles (Miller and Conko 2001). But decisions about how much evidence to demand before taking regulatory actions necessarily incorporate both scientific judgments and value judgments. Because the scientific conventions for inferring evidence of harm in some fields might require placing the public at risk for extended periods of time before the evidence could be accumulated, precautionary decisions to engage in particular forms of regulation may sometimes be appropriate in response to more limited evidence (Cranor 2011; Martuzzi 2007).

*Financial, personal, and cultural influences*. A second reason to avoid trying to maintain a sharp distinction between science and values in the policy context is that personal, ethical, political, and cultural values unavoidably influence scientific reasoning. This point is illustrated by recent conceptual and empirical literature on the ways that financial relationships can affect scientific judgment and reasoning (Dana and Loewenstein 2003; Elliott 2008; Resnik and Elliott 2013). An investigative report found that 17 of the 18 authors of the initial editorial by Dietrich et al. (2013) had ties to regulated industries (Horel and Bienkowski 2013). In response to this investigation, Dietrich replied, “[w]e do not believe the discussion on the conflicts of interests will serve anybody because it takes away the focus from the real issue” (quoted by Horel and Bienkowski 2013). Bas Blaauboer, another co-author of the editorial by Dietrich et al. (2013), insisted that it was “very stupid” to think that his industry involvement influenced his opinion (quoted by Horel and Bienkowski 2013). But psychological studies have suggested that financial interests can exert subconscious influences on human judgment even when individuals are instructed about those potential influences and motivated to remain objective (Babcock et al. 1997; Dana and Loewenstein 2003; Orlowski and Wateska 1992). Importantly, people typically underestimate the strength of these influences (Dana and Loewenstein 2003; Katz et al. 2003). Some commentators also worry that interest groups with “deep pockets” can use their financial and political power to skew public debate by magnifying the influence of sympathetic scientists through strategic funding efforts and public relations campaigns (McGarity and Wagner 2008; Michaels 2008).

Financial relationships are not the only factors that can influence scientists. Studies of risk perception have found that even among professional toxicologists, men tend to have systematically lower perceptions of chemical risks than women, and those employed by industry have lower perceptions of risk than those in academic settings (Slovic et al. 1997). Some of these employment effects may be caused or exacerbated by the phenomenon of group polarization, whereby people exposed primarily to those who share similar views ultimately adopt more extreme positions (e.g., Sunstein 2005). Even cultural values—concerns about equality, authority, individualism, and community—can influence individuals’ risk perceptions (Kahan 2010). This evidence from the social sciences suggests that although scientists can strive for objectivity, it is unrealistic to think that they can provide policy advice without being influenced by a variety of subconscious factors, such as interests and values.

## Conclusions

Given both that values play a crucial role in choosing standards of evidence in the policy context and that values have subconscious influences on scientific judgment that are impossible to eliminate completely, we suggest that the best way to do policy-relevant research is for scientists to be as transparent as possible about the ways in which interests and values may influence their work (Ashford 1988). The analytic–deliberative approach to risk characterization described in *Understanding Risk* (NRC 1996) provides one model for promoting this sort of transparency, but a number of other strategies for promoting transparency are also available. For example, efforts to incorporate scientists from a range of different stakeholder groups on government advisory bodies can help to uncover and elucidate implicit value judgments in science advice and promote democratic decision making (Resnik 2009). These efforts to uncover implicit value judgments are important, given that values can influence subtle decisions about research questions, methodologies, terminology, and models (Ashford 1988; Douglas 2009; Elliott 2011; Kriebel et al. 2001). Disclosures of competing financial interests and nonfinancial interests (such as professional or political allegiances) also provide opportunities for more transparent discussions of the impact of potentially implicit and subconscious values (Resnik and Elliott 2013).

When scientists are aware of important background assumptions or values that inform their work, it is valuable for them to make these considerations explicit. They can also make their data publicly available and strive to acknowledge the range of plausible interpretations of available scientific information, the limitations of their own conclusions, the prevalence of various interpretations across the scientific community, and the policy options supported by these different interpretations. This approach has much in common with Ashford’s seminal call for scientists to be transparent about their values (Ashford 1988) and with Pielke’s vision of scientists as “honest brokers” who open up discussions about the range of options available to decision makers (Pielke 2007). It may even be valuable for scientists to reflect on how their work fits into broader social frames or narratives so that they can anticipate how their claims are likely to be misinterpreted or used to promote particular political or economic agendas (McKaughan and Elliott 2013).

Although scientists are rightly taught to strive for objectivity, efforts to maintain a sharp distinction between science and policy are likely to be counterproductive in such cases as the recent dispute over EDCs. When scientific evidence is disputed and major regulatory decisions are at stake, it is unrealistic to think that scientists will not be influenced by their financial, social, political, and personal interests or values when they offer advice to policy makers. Moreover, judgments about whether EDCs exhibit thresholds or whether an alleged EDC will have adverse effects in humans rest not only on scientific evidence but also on value-laden judgments about the appropriate standards of evidence. Even calls for decisions “based on sound science” incorporate implicit value judgments about the appropriate standards of evidence for drawing policy-relevant conclusions. In cases such as these, efforts to suppress or hide interests or values may actually damage scientific objectivity and public trust, whereas a willingness to bring implicit interests or values into the open may be the best path to promoting good science and policy.

## References

[r1] Ashford N (1988). Science and values in the regulatory process.. Stat Sci.

[r2] Babcock L, Loewenstein G, Issacharoff S (1997). Creating convergence: debiasing biased litigants.. Law Soc Inquiry.

[r3] Bergman Å Andersson AM Becher G van den Berg M Blumberg B Bjerregaard P 2013 Science and policy on endocrine disrupters must not be mixed: a reply to a “common sense” intervention by toxicology journal editors [Editorial] Environ Health 12 69; 10.1186/1476-069X-12-69 23981490 PMC3765603

[r4] Cranor CF. (1993).

[r5] Cranor CF. (2008).

[r6] Cranor CF. (2011). Legally Poisoned: How the Law Puts Us at Risk from Toxicants.

[r7] Cressey D 2013 Journal editors trade blows over toxicology. Nature; 10.1038/nature.2013.13787 [Online 20 September 2013]

[r8] Dana J, Loewenstein G (2003). A social science perspective on gifts to physicians from industry.. JAMA.

[r9] Dietrich DR von Aulock S Marquardt H Blaauboer B Dekant W Kehrer J 2013 Scientifically unfounded precaution drives European Commission’s recommendations on EDC regulation, while defying common sense, well-established science and risk assessment principles [Editorial] Food Chem Toxicol 62 A1 A4 23835284 10.1016/j.fct.2013.07.005

[r10] Douglas HE. (2009). Science, Policy, and the Value-Free Ideal.

[r11] EC (European Commission). (2013). Commission Recommendation of XXXX: Defining Criteria for Endocrine Disruptors.. http://www.environmentalhealthnews.org/ehs/news/2013/pdf-links/2013.06.11%20EDC_Recommendation%20Commission%20Draft.pdf.

[r12] Elliott KC (2008). Scientific judgment and the limits of conflict-of-interest policies.. Account Res.

[r13] Elliott KC. (2011).

[r14] Gore AC Balthazart J Bikle D Carpenter DO Crews D Czernichow P 2013 Policy decisions on endorine disruptors should be based on science across disciplines: a response to Dietrich et al. [Editorial] Endocrinology 154 3957 3960 24048095 10.1210/en.2013-1854 PMC5398595

[r15] Grandjean P Ozonoff D 2013 Transparency and translation of science in a modern world [Editorial] Environ Health 12 70; 10.1186/1476-069X-12-70 23981514 PMC3765922

[r16] Horel S, Bienkowski B. (2013). Special report: scientists critical of EU chemical policy have industry ties. Environ Health News.. http://www.environmentalhealthnews.org/ehs/news/2013/eu-conflict.

[r17] IPCS (International Programme on Chemical Safety). (2002). Global Assessment of the State-of-the-Science of Endocrine Disruptors. Geneva:World Health Organization, IPCS.. http://www.who.int/ipcs/publications/new_issues/endocrine_disruptors/en/.

[r18] Kahan D (2010). Fixing the communications failure.. Nature.

[r19] Katz D, Caplan A, Merz J (2003). All gifts large and small: toward an understanding of the ethics of pharmaceutical industry gift-giving.. Am J Bioeth.

[r20] Kriebel D, Tickner J, Epstein P, Lemons J, Levins R, Loechler EL (2001). The precautionary principle in environmental science.. Environ Health Perspect.

[r21] Lehman-McKeeman L Kaminski N 2013 The hazards of playing it safe: perspectives on how the Society of Toxicology should contribute to discussions on timely issues of human and environmental safety [Editorial] Toxicol Sci 136 1 3

[r22] Longino HE. (1990). Science as Social Knowledge: Values and Objectivity in Scientific Inquiry.

[r23] Martuzzi M (2007). The precautionary principle: in action for public health.. Occup Environ Med.

[r24] McGarity TO, Wagner W. (2008). Bending Science: How Special Interests Corrupt Public Health Research.

[r25] McKaughan DJ, Elliott KC (2013). Backtracking and the ethics of framing: lessons from voles and vasopressin.. Account Res.

[r26] Michaels D. (2008).

[r27] Miller HI, Conko G (2001). Precaution without principle.. Nat Biotechnol.

[r28] NRC (National Research Council). (1996). Understanding Risk: Informing Decisions in a Democratic Society.

[r29] Orlowski J, Wateska L (1992). The effects of pharmaceutical firm inticements on physician prescribing patterns.. Chest.

[r30] Pielke RA. (2007). The Honest Broker: Making Sense of Science in Policy and Politics.

[r31] Resnik DB. (2007).

[r32] Resnik DB. (2009).

[r33] Resnik DB, Elliott KC (2013). Taking financial relationships into account when assessing research.. Account Res.

[r34] Slovic P, Malmfors T, Mertiz C, Neil N, Purchase I (1997). Evaluating chemical risks: results of a survey of the British Toxicology Society.. Hum Exp Toxicol.

[r35] Sunstein C. (2005).

